# Thyroid Hormones Play Role in Sarcopenia and Myopathies

**DOI:** 10.3389/fphys.2018.00560

**Published:** 2018-05-23

**Authors:** Flavia F. Bloise, Thamires S. Oliveira, Aline Cordeiro, Tania M. Ortiga-Carvalho

**Affiliations:** Laboratory of Translational Endocrinology, Carlos Chagas Filho Institute of Biophysics, Federal University of Rio de Janeiro, Rio de Janeiro, Brazil

**Keywords:** thyroid hormone, skeletal muscle, aging, sarcopenia, myopathy, weakness, myalgia

## Abstract

Skeletal muscle maintains posture and enables movement by converting chemical energy into mechanical energy, further contributing to basal energy metabolism. Thyroid hormones (thyroxine, or T4, and triiodothyronine, or T3) participate in contractile function, metabolic processes, myogenesis and regeneration of skeletal muscle. T3 classically modulates gene expression after binding to thyroid hormone nuclear receptors. Thyroid hormone effects depend on nuclear receptor occupancy, which is directly related to intracellular T3 levels. Sarcolemmal thyroid hormone levels are regulated by their transport across the plasma membrane by specific transporters, as well as by the action of deiodinases types 2 and 3, which can activate or inactivate T4 and T3. Thyroid hormone level oscillations have been associated with the worsening of many myopathies such as myasthenia gravis, Duchenne muscular dystrophy (DMD) and rhabdomyolysis. During aging skeletal muscle show a decrease in mass and quality, known as sarcopenia. There is increasing evidence that thyroid hormones could have a role in the sarcopenic process. Therefore, in this review, we aim to discuss the main effects of thyroid hormones in skeletal muscular aging processes and myopathy-related pathologies.

## Introduction

Skeletal muscle (SM) maintains posture and enables movement by converting chemical energy into mechanical energy, further contributing to basal energy metabolism (Schiaffino and Reggiani, 2011; Frontera and Ochala, 2015). The SM architecture is characterized by a particular arrangement of muscle fibers that are multinucleated (Frontera and Ochala, 2015). SM fibers are classified as slow or fast twitch fibers: 1, 2a, 2x, and 2b, named in order of increasing speed of contraction and overall decreasing ATP-generating capacity. The expression of myosin heavy chain isoforms also characterizes muscle fibers: type 1 express myosin-7 (MYH7), type 2a express myosin-2 (MYH2), type 2x express myosin-1(MYH1), and type 2b express myosin-4 (MYH4) (Schiaffino and Reggiani, 2011). The SM metabolism and fibers type are regulated by innervation and soluble factors, such as thyroid hormones (THs) (Schiaffino and Reggiani, 2011; Bloise et al., 2018).

TH serum levels are controlled by the hypothalamic-pituitary-thyroid (HPT) axis (Ortiga-Carvalho et al., 2016). However, the nutritional status, illness and aging can change the set points of the HPT axis, leading to changes in TH synthesis by the thyroid gland (Fliers et al., 2015). Thyroxin (T4) is the hormone most abundantly produced by the thyroid. However, triiodothyronine (T3) is considered the active form of the TH, since it binds to thyroid hormone receptors (THRs) as part of the classical pathway (genomic actions) (Ortiga-Carvalho et al., 2016). THRs are members of the nuclear hormone receptor superfamily and are encoded by two genes: *Thra* and *Thrb* (Cheng et al., 2010), THRα1 is the most abundant THR isoform expressed in SM (Bloise et al., 2018). The biological activity of THs via THRs is determined by the intracellular levels of THs, which are dependent on several factors, such as serum TH concentration, membrane transporters and the activity of deiodinase enzymes (Ortiga-Carvalho et al., 2016). Monocarboxylate transporter 8 (MCT8) is the main TH transporter in SM. The deiodination of T4 into T3 can occur inside the cell by the action of deiodinase type 2 (D2) or on the plasma membrane by the action of deiodinase type 1 (D1). D1 and deiodinase type 3 (D3) also catalyze the conversion of T4 into reverse T3 (rT3). Furthermore, D3 converts T3 into 3,3’-T2. SM expresses D2 and D3. The production of T3 from T4 is catalyzed by D2, increasing the intracellular availability of T3 and nuclear receptor binding, while the inactivation of T4 and T3 is the main function of D3 (Salvatore, 2018).

T3 is probably the most studied TH regarding muscle physiology; however, other THs and derivatives also influence muscle physiology. High levels of T4 reduce contractility in the canine diaphragm, the primary respiratory SM (Miyashita et al., 1992). Additionally, it was demonstrated that the TH metabolite 3,5-T2 rapidly stimulates respiratory chain activity, mitochondrial thermogenesis, and fatty acid oxidation in SM cells (Lombardi et al., 2009). However, the exact mechanisms triggered by T2 are still unclear. Considering the importance of T3 and the large amount of data on this issue, we will mainly focus our analysis on T3 effects.

T3 stimulates *Myh1*, *Myh2*, *Myh4* expression and represses *Myh7* gene expression (Bloise et al., 2018). Additionally, the control of intracellular T3 levels is crucial for the progression of myogenesis (Ambrosio et al., 2017; Salvatore, 2018). SM mitochondrial function is also modulated by T3 (Bloise et al., 2018). In aged subjects, the serum T3 levels decrease. The reduction of THs is one factor involved in the aging process. During aging, myogenesis is reduced, SM metabolism is modulated and the type II fibers are decreased (Snijders et al., 2015). Thus, the decrease in TH signaling seems to influence SM during aging.

## Thyroid Hormone and Sarcopenia

Aging is a multifactorial process that involves changes in serum hormone levels, mitochondrial dysfunction, accumulation of cellular damage, low-grade chronic inflammation and altered tissue function (Nikoletopoulou et al., 2014; Goljanek-Whysall et al., 2016). The serum TH concentration decreases with aging, and it has been correlated with age-associated pathologies (da Costa et al., 2001). SM is also affected by age because men and women older than 60 years present decreased muscle quality (Doherty, 2003; Collino et al., 2013; Dumont et al., 2015a). The age-related loss of muscle mass, strength and quality is called sarcopenia. Like aging, sarcopenia is multifactorial and associated with decreases in myogenesis and alterations in biochemical profiles and fiber types (Doherty, 2003; Collino et al., 2013; Dumont et al., 2015a).

Muscle cross-sectional area decreases with aging (Doherty, 2003; Schiaffino and Reggiani, 2011). In older men, the reduction of muscle mass is associated with a decrease in type 2b fibers and minimal changes in the type 1 fiber profile (Schiaffino and Reggiani, 2011; Nilwik et al., 2013). Animal studies corroborate the fiber type change associated with aging. Aged rats present a transition of fast fiber types into slower ones, including decreases in type 2b fibers, increases in type 2x fibers and conversion of type 2a fibers to type 1 fibers (Larsson et al., 1991, 1993; Schiaffino and Reggiani, 2011). The change in fiber type profile is associated with alterations in neuromuscular junctions (Goljanek-Whysall et al., 2016); however, the causal association between the decrease in motor unit function and the development of sarcopenia is still not totally clear (Ling et al., 2009; Pannérec et al., 2016; Piasecki et al., 2016). Other factors are associated with the loss of muscle quality; thus, the decrease in muscle mass, strength and power is multifactorial. It is important to note that THs induce the transition from a slower fiber type into a faster one and could be involved in the sarcopenic process (Simonides and van Hardeveld, 2008; Salvatore et al., 2014). T3 administration to old rats increased *Myh2* and *Myh1* expression in the slow-twitch soleus; however, no significant changes were observed in the fast-twitch extensor digitorum longus after the treatment (Larsson et al., 1995). Muscle cross-sectional area is smaller in elderly subclinical hypothyroid patients compared to euthyroid age-matched controls (Moon et al., 2010). Additionally, treatment of these patients to a euthyroid state improves muscle strength and cross-sectional area (Brennan et al., 2006). However, the overall muscle quality of elderly subclinical hypothyroid and euthyroid patients is similar (Moon et al., 2010).

During aging, a decrease in ATP production and mtDNA in muscle fibers is observed (Short et al., 2005; Carter et al., 2015; Li et al., 2016). Overexpression of the mitochondrial THRA isoform, p43, stimulates mitochondrial respiration and induces an oxidative phenotype in SM (Casas et al., 2009). However, the overexpression of p43 increased oxidative stress and decreased mitochondrial content in the muscles of 6-, 11-, and 23-month-old mice (Casas et al., 2009). Despite the evidence pointing to a possible causal relationship between THs and sarcopenia during aging, more studies are necessary to understand the molecular and physiological mechanism of this association.

## Thyroid Hormones and Myopathies

Hyperthyroid patients present muscle weakness and increased Ca^2+^ recycling (Riis et al., 2005; Brennan et al., 2006), and hyperthyroid mice showed increased muscle fatigability compared to control groups (Elnakish et al., 2015). Exercise intolerance, cramps, stiffness, muscle pain (myalgia) and muscle weakness are common symptoms in hypothyroid and hyperthyroid patients. Moreover, Allan–Herndon–Dudley syndrome patients (caused by *Slc16a2* mutation, the gene encoding MCT8) have decreased muscle mass and tone. In *Slc16a2-Knock-out* mice (*Mct8KO)* the decreases in muscle mass and tone seem to be associated with increased muscular TH content (Maranduba et al., 2006; Di Cosmo et al., 2013). Additionally, some rare muscular myopathies are associated with changes in thyroid status, such as myasthenia gravis, Duchenne muscular dystrophy (DMD) and rhabdomyolysis (Fukazawa et al., 1990; McIntosh et al., 1994; McArdle et al., 1998; Vincent et al., 2012; Gilhus et al., 2015; Sindoni et al., 2016). A summary of myopathies and TH physiology is presented in **Table 1**.

**Table 1:** Summary of myopathies and TH physiology.

Pathology	SM alteration	TH physiology	Sarcolemmal TH levels	Species	Reference
Allan–Herndon–Dudley syndrome	↓ SM mass and tone	MCT8 deficiency; decreased T4 and increased T3 levels	T3 increased	Mice	Maranduba et al., 2006; Di Cosmo et al., 2013.
Hypothyroid myopathy	myalgia, myoedema, exercise intolerance, cramps, stiffness, increased levels of creatine kinase	Decreased T4 and T3 levels	Not reported	Human	Rodolico et al., 1998; Duyff et al., 2000; Monici et al., 2002; Sindoni et al., 2016.
Myasthenia gravis	muscle weakness and fatigability	Increased hyperthyroidism in patients	Not reported	Human	Osserman et al., 1967; Puvanendran et al., 1979; Vincent et al., 2012; Gilhus et al., 2015.
Duchenne muscular dystrophy	tissue injury, reduced SM repair	Similar to euthyroid population	Not reported	Human and mice	Petrof et al., 1993; McIntosh and Anderson, 1995; McArdle et al., 1998; Yiu and Kornberg, 2015.
Mutation in *SECISBP2*	muscle weakness, satellite cell loss and progressive peripheral myopathy	Atypical resistance to THs, increased rT3 and T4 levels and decreased T3 levels	Not reported	Human	Schweizer and Fradejas-Villar, 2016.
SPEN1-related myopathy	muscle atrophy predominantly affecting axial muscles, muscle weakness	Not reported	Not reported	Human and mice	Arbogast and Ferreiro, 2010; Castets et al., 2011; Schweizer and Fradejas-Villar, 2016.
Myotonic dystrophy	muscle weakness, myotonia; DM1: atrophy in type 1 fibers; DM2: atrophy in type 2 fibers	20% of patients with DM have thyroid diseases, and 32% of DM2 patients present thyroid dysfunction	Not reported	Human	Vihola et al., 2003; Montagnese et al., 2017.
Rhabdomyolysis	muscle pain, weakness	Low serum T3 levels seem to aggravate the disease	Not reported	Human	Ardalan et al., 2011; Cai and Tang, 2013; Tatar et al., 2014.


Hypothyroid myopathy is a collection of muscle symptoms that can be present in the hypothyroid condition (Sindoni et al., 2016). These symptoms include myalgia, muscular pseudohypertrophy (myoedema), exercise intolerance, cramps and stiffness. Hypothyroid myopathy could be associated with the reduction in muscle energy metabolism and less efficient contraction-relaxation rates due to the reduction of intramuscular T3 levels to bellow optimal (McDaniel et al., 1977; Sindoni et al., 2016). Additionally, 57-90% of hypothyroid patients have increased levels of creatine kinase (CK), a marker of muscle damage (Hekimsoy and Oktem, 2005). In hypothyroid patients, the CK levels fall after L-T4 replacement therapy (Rodolico et al., 1998; Duyff et al., 2000).

Additionally, hyperthyroidism is also a risk factor for other myopathies, such as myasthenia gravis, which is an autoimmune disease mediated by autoantibodies that target neuromuscular junctions (Gilhus et al., 2015). Acetylcholine receptors are the primary target of these autoantibodies, leading to muscle weakness and fatigability (Vincent et al., 2012; Gilhus et al., 2015). Elevated serum T3 levels, as seen in hyperthyroidism, can induce B cell activation and plasma cell antibody secretion in the absence of antigens (Bloise et al., 2014). Like the majority of autoimmune diseases, myasthenia gravis is associated with a genetic predisposition, environmental factors and hormonal levels (Berrih-Aknin, 2014; Yeh et al., 2015). Thus, high serum T3 levels in susceptible patients could lead to autoantibody production. Hyperthyroidism is more common in myasthenia gravis patients than in the general population (Osserman et al., 1967; Puvanendran et al., 1979). The treatment of thyrotoxicosis improved muscle response in myasthenia gravis patients (Puvanendran et al., 1979). Furthermore, administration of THs to euthyroid patients worsened myasthenia gravis symptoms (Eaton, 1947; Grob and Harvey, 1953). In this context, high serum levels of THs seem to precede autoimmune disease (Rodrigué et al., 1989; Appenzeller et al., 2009). Taken together, we speculate that THs are associated with the initial development of myasthenia gravis.

DMD is a debilitating hereditary disease. The progression of the disease is rapid, and patients usually die between the 2nd and 3rd decade of life from respiratory and cardiac failure (Yiu and Kornberg, 2015). The *DMD* gene encodes dystrophin, which is absent or non-functional in DMD subjects (Aartsma-Rus et al., 2016). Normal muscular contraction leads to tissue injury in DMD patients (Petrof et al., 1993). Additionally, dystrophin is also involved in the asymmetric division of satellite cells (Dumont et al., 2015b). Thus, factors that influence muscle satellite cell differentiation and proliferation can affect the pathological outcome. The local levels of THs are finally regulated through the myogenic process (Ambrosio et al., 2017). D3 is highly expressed in early activated muscle stem cells. The inactivation of THs is essential for the survival and expansion of the satellite cell pool. Later, an increase in intracellular T3 levels by D2 is critical for progenitor cell differentiation (Ambrosio et al., 2017). Thus, the balance in intracellular T3 levels controls myogenesis by influencing progenitor cell proliferation and differentiation (Bloise et al., 2018). Mdx mice are a DMD model because they do not express dystrophin. Mdx satellite cells have reduced myogenic potential (Biressi et al., 2014). Induction of hypothyroidism in mdx mice leads to a reduction in the size of regenerated fibers after crush injury compared to fibers in euthyroid mdx mice (McIntosh et al., 1994). Additionally, central nuclei fibers, indicative of an early stage of muscle regeneration, and serum CK levels are reduced in hypothyroid mdx mice compared to euthyroid mdx mice (McArdle et al., 1998). However, the number of mononuclear cells is increased in hypothyroid mdx compared to euthyroid mdx, and delayed muscle necrosis is observed (McIntosh and Anderson, 1995; McArdle et al., 1998). Although, hyperthyroidism did not change the fiber size in mdx animals and did decrease fiber size in wild-type animals (Pernitsky et al., 1996). Additionally, dystrophy is more severe in slow-twitch SM in hyperthyroid mdx mice (Anderson et al., 1994; Pernitsky et al., 1996). Thus, we can speculate that TH signaling increases satellite cell numbers but does not improve fiber regeneration in DMD.

The selenoproteins are a group of 25 proteins that contains the amino acid selenocysteine (Schweizer and Fradejas-Villar, 2016). The only two well characterized human selenoprotein groups are glutathione peroxidases and deiodinases. Human mutations in deiodinases (*DIO* gene) are not known. However, Thr92Ala polymorphisms in the D2 gene (*DIO2*) lead to a decrease in SM D2 activity and seem to be associated with decreased insulin sensitivity in this tissue (Canani et al., 2005; Dora et al., 2010). Additionally, a mutation in *SECISBP2* leads to an atypical resistance to THs, elevated rT3 and T4 levels and a decreased T3 levels. Patients with this mutation present decreased D1 and D2 activity, and some patients can present muscle weakness and progressive peripheral myopathy (Schweizer and Fradejas-Villar, 2016). However, only a mutation of selenoprotein N (SelN) leads to a well characterized myopathy (Schweizer and Fradejas-Villar, 2016). SelN is ubiquitously expressed and seems to be involved in Ca^+2^ homeostasis and protection against oxidative stress (Pitts and Hoffmann, 2018). The SPEN1-related myopathy is a severe neuromuscular disorder caused by a deficiency of SelN and is characterized by respiratory insufficiency, muscle atrophy that predominantly affects axial muscles, and muscle weakness (Arbogast and Ferreiro, 2010). As with DMD, the SPEN1-related myopathy is associated with satellite cells loss (Castets et al., 2011). A *Sepn1*-knock-out mouse model shows impaired muscle regeneration and is more sensitive to challenging physical activity (Rederstorff et al., 2011). Physical exercise increases skeletal muscle D2 activity, and thus, it seems to increase sarcolemmal T3 levels (Bocco et al., 2016). The increased sensitivity to physical challenge in *Sepn1*-knock-out mice could be associated with a failure in recovering from muscle damage and not to a decrease in TH responsiveness during exercise. The pathogenesis of SPEN1-related myopathy still not completely understood; however, the control of embryonic myogenesis is a key point, and THs could be involved in the regulatory process.

Myotonic dystrophy (dystrophia myotonica, DM) is an autosomal dominant monogenic disorder and is the predominant muscular dystrophy in adults (Fitzgerald et al., 2016). Myotonic dystrophy 1 and 2 (DM1, DM2) are caused by unstable CTG or CCTG repeats within the gene *DMPK* or *CNBP* (*ZNF9*), respectively (Thornton, 2014). The diseases are characterized by cataracts, muscle weakness, myotonia, and adult-onset multisystem degenerative disorder. Patients’ symptoms are very heterogeneous, and thus, DM1 patients are commonly classified as having congenital DM1, childhood DM1, classical DM1 or minimal DM1 in order to improve treatment. The latter two occur predominantly during adult life as DM2 (Thornton, 2014). DM1 patients show selected atrophy of type 1 fibers (Vihola et al., 2003), while DM2 patients show selective atrophy of type 2 fibers (Vihola et al., 2003). Alteration in the TH axis is a common comorbidity in DM1 and DM2 patients; 20% of DM patients have thyroid diseases, and 32% of DM2 patients present thyroid dysfunction (Montagnese et al., 2017). DM1 patients present decreases in serum free T3 levels and reduced iodine uptake by the thyroid (Fukazawa et al., 1990). Low-dose TH replacement has been used to decrease pain in DM2 patients. Thus, the decrease in TH levels could be associated with the muscle atrophy and weakness present in DM.

Rhabdomyolysis is a life-threatening syndrome caused by any damage to SM tissue that results in sarcolemmal membrane rupture and leakage of intracellular components. Rhabdomyolysis is characterized by muscle pain, weakness, a large increase in CK levels and pigmenturia (Chavez et al., 2016). Low T3 serum levels seem to aggravate the disease (Ardalan et al., 2011; Cai and Tang, 2013). However, hypothyroidism is a non-traumatic and uncommon cause of this disease. Therefore, the exact association between rhabdomyolysis and hypothyroidism is still not clear (Satarasinghe et al., 2007; Tatar et al., 2014; Sindoni et al., 2016). The influence of THs on mitochondrial activity seems to be a possible correlation. Additionally, patients with acute kidney disease have a higher risk of developing rhabdomyolysis related to hypothyroidism (Cai and Tang, 2013). Kidney dysfunction by itself could induce the reduction in serum TH levels because kidneys are associated with systemic TH clearance. Moreover, chronic kidney disease patients normally present low serum T3 levels as a result of this critical disease.

SM function is regulated by a complex system involving neuroendocrine signaling. Herein, we present evidence linking THs to the development of and complications observed in myopathies. Additionally, the collective changes observed in SM with aging are similar to alterations associated with a decrease in TH signaling. However, we do not know if changes in TH status are a cause or consequence of the SM- related disease. Thus, more studies must be performed to better understand the interconnection between THs and SM. The administration of THs or THRa agonists could be use in elderly patients as a future treatment to manage sarcopenia. Meanwhile, more studies have to be performed to understand the safety and possible beneficial actions of these treatments.

## Author Contributions

FB and TO-C participated in fund acquisition. All authors participated in the conception and writing of the manuscript, reviewing the literature, and critically analyzing the text.

## Conflict of Interest Statement

The authors declare that the research was conducted in the absence of any commercial or financial relationships that could be construed as a potential conflict of interest.
